# Metabolic impact of reduced-protein Nordic diet-based complementary feeding: a secondary analysis of a randomized controlled study^☆^

**DOI:** 10.1016/j.ajcnut.2026.101238

**Published:** 2026-02-16

**Authors:** Xuan He, Zhichao Zhang, Ulrica Johansson, Daniel J Tancredi, Olle Hernell, Bo Lönnerdal, Torbjörn Lind, Carolyn M Slupsky

**Affiliations:** 1 Department of Nutrition, University of California-Davis, Davis, CA, United States; 2 Department of Food Science and Technology, University of California-Davis, Davis, CA, United States; 3 Department of Clinical Sciences, Pediatrics, Umeå University, Sweden; 4 Department of Pediatrics, School of Medicine, University of California-Davis, Davis, CA, United States

**Keywords:** infant, complementary feeding, Nordic diet, metabolomics, Early Protein Hypothesis, plant-based food, structural equation modeling

## Abstract

**Background:**

Protein intake among children in resource-rich countries often exceeds current recommendations. Higher protein consumption during infancy has been associated with an increased risk of obesity later in life.

**Objectives:**

We conducted a secondary analysis of a randomized controlled trial where 250 infants were randomly assigned to receive either a protein-reduced Nordic diet or a conventional Swedish complementary diet. Our aims were to examine the metabolic responses to the intervention using plasma metabolomics and to test the pathways proposed by the Early Protein Hypothesis by linking cumulative protein intake to circulating branched-chain amino acids (BCAAs), insulin-like growth factor-1 (IGF-1), and growth outcomes using structural equation modeling (SEM).

**Methods:**

Targeted proton nuclear magnetic resonance (^1^H-NMR) metabolomics was performed on plasma samples collected at 12 and 18 mo. Two SEM models were constructed: one modeled body weight and the other modeled BMI as growth outcomes using data collected up to age 18 mo. Model fit indices were assessed, and path diagrams were used to visualize relationships.

**Results:**

The Nordic diet led to reduced-protein intake and a distinct infant plasma metabolomic profile. Circulating BCAAs and their catabolites were significantly lower in the reduced-protein Nordic diet compared with the conventional diet. Cumulative protein intake positively correlated with plasma IGF-1 concentrations {weight-based SEM: β = 0.40 [95% confidence interval (CI): 0.03, 0.48]; BMI-based SEM: β = 0.43 [95% CI: 0.05, 0.50]}. Plasma total BCAAs were positively associated with plasma IGF-1 levels [weight-based SEM: β = 0.16 (95% CI: 0.00, 0.76); BMI-based SEM: β = 0.17 (95% CI: 0.02, 0.78)]. After accounting for metabolite-mediated effects on IGF-1 and insulin, cumulative protein intake remained significantly associated with infant body weight [β=0.36 (95% CI: 0.02, 0.99)], but not BMI.

**Conclusions:**

Complementary feeding during infancy substantially shapes the plasma metabolome. Reducing protein intake from complementary feeding helps attenuate rapid infant weight gain, a well-established early-life predictor of later obesity.

## Introduction

Early-life nutrition is crucial for supporting optimal growth and development and establishing a foundation for long-term health [1,2]. Complementary feeding begins when breastmilk and/or infant formula alone no longer meet nutritional requirements and typically starts at the age of 4 to 6 mo and continues until 2 y [3]. Exactly which foods are best for optimal growth and development during this period is not universally agreed upon, as complementary feeding practices are deeply influenced by cultural beliefs and family traditions. To this end, there has been a global effort to develop national food-based dietary guidelines that integrate health, environmental sustainability, and sociocultural aspects by promoting regional foods and eating traditions [[4], [5], [6]]. For example, in the Nordic and Baltic countries, the Nordic Nutrition Recommendations (NNR) [7] emphasize higher intakes of regionally produced and seasonal foods that are low in saturated fats as well as added sugar and salt and include fruits, vegetables, legumes, whole grains, and fish, while limiting the consumption of red and processed meats, sweetened beverages and sweets [8,9]. Although this dietary pattern has been linked to reduced risk of noncommunicable diseases [10,11], the integration of the Nordic diet into complementary feeding for infants has not been extensively evaluated in randomized controlled trials.

High protein intake during infancy has been linked to an increased risk of obesity later in life [12,13]. Specifically, high protein infant formula given before age 6 mo and high animal and dairy protein intake at 12 mo have been associated with elevated circulating insulin-like growth factor-1 (IGF-1) and higher body weight during infancy, and higher BMI in later childhood. [[14], [15], [16], [17]]. This proposed link between higher protein intake in early life and increased long-term obesity risk is referred to as the Early Protein Hypothesis. Current dietary recommendations for infants and children advocate for a lower protein intake than previous guidelines suggested, with a growing recognition of the importance of protein quality [18]. In developed countries, protein intake among children and adolescents has been reported to be 2 to 3 times higher than currently recommended, with protein predominantly derived from animal sources [19,20]. A recent survey in Sweden revealed that >80% of infants at one year of age consumed more protein than is currently recommended [21].

To understand the impact of complementary feeding on infant metabolism, we conducted a secondary analysis of data from a randomized controlled trial where 250 infants were assigned to either a protein-reduced, fruits and vegetable enriched, Nordic diet portfolio-based complementary food (Nordic diet) group, or a conventional diet representing typical Swedish complementary feeding practices (conventional diet) group from baseline (4 to 6 mo) to age 18 mo. Previously, we reported good acceptance of the provided food and a high adherence to the intervention diet. Overall, infants in the Nordic diet group achieved a 26% reduction in protein intake at 9 mo, 29% at 12 months, and 17% at 18 months [22,23]. These dietary differences were associated with significant reductions in serum urea and IGF-1 levels in the Nordic group, with no significant effects on growth outcomes and body composition at 12 and 18 mo [22].

Here, we report on the infant plasma metabolome at age 12 and 18 mo to examine the impact of the reduced-protein Nordic diet on metabolism. We hypothesized that lower protein would alter the plasma metabolome by decreasing branched-chain amino acid (BCAA) concentrations, which are known to exhibit an insulinogenic effect and have potential to increase IGF-1 and subsequent growth. Building on this metabolic rationale, we aimed to validate the Early Protein Hypothesis by leveraging detailed dietary records, anthropometric measurements, body composition, biochemical analyses, and metabolomics data from this cohort. Specifically, we applied structural equation modeling (SEM) to investigate the complex relationship between protein intake and circulating BCAAs, IGF-1, and insulin levels, as well as infant growth outcomes.

## Methods

### Participants and study design

The OTIS (Swedish acronym for Optimized Complementary Feeding) study is a randomized controlled trial with a multicomponent design, conducted in Umeå, Sweden, from April 2015 to January 2018. A total of 250 healthy Swedish infants were recruited and randomly assigned to 1 of 2 groups: the Nordic diet group (intervention, *n* = 125) or the conventional diet group (control, *n* = 125). At the time of recruitment, none of the infants had started complementary feeding. These infants were then introduced to different complementary feeding regimens from baseline (4 to 6 mo), and these diets were continued until age 18 mo. Participating families were contacted monthly to ensure adherence to the protocol, resolve any study-related issues, assess the consumption, supply study products, and review any reported symptoms. Study visits were conducted at the Pediatric Research Unit at the Department of Clinical Sciences, Pediatrics, Umeå University Hospital, scheduled at enrollment, 9, 12, and 18 mo of age. All participating families, regardless of group assignment, received comprehensive services from the local community child health care centers, which included vaccination, growth monitoring, as well as general health and nutrition advice at no cost.

The primary outcome of this study was the assessment of body composition via deuterium oxide (D_2_O) dilution method for which results have been previously published [22]. Details on other secondary outcomes have also been published [[22], [23], [24]]. The present analysis focused on plasma metabolomics outcomes at 12 and 18 mo of age, which were not reported earlier.

Details on methodology, such as inclusion and exclusion criteria, estimation of sample size, randomization, and blinding techniques, were previously published [25]. Briefly, infants were stratified by sex and randomly assigned to one of the complementary feeding regimens using a computerized tool in blocks of 10. Blinding in the study was partially implemented. Parents, research nurses, and dieticians were aware of the group allocations because food items and the menu were visually recognizable. All other personnel, including the laboratory personnel and researchers conducting dietary analyses, were fully blinded to participants’ group allocations.

Eligible participants were healthy, singleton infants aged 4 to 6 mo at inclusion, born at ≥37 wk of gestation, with a birth weight >2.5 kg. Participating families were required to live in Umeå and plan to stay there throughout the 12-month study period, without starting any external childcare services. Exclusion criteria included use of supplements or medications and any chronic illnesses that could affect feeding or study outcomes, such as food allergies or intolerances to study products. Additional exclusion criteria included the initiation of complementary feeding at the time of recruitment, iron deficiency, or any other biochemical abnormalities requiring medical intervention, as determined by the study physician.

Participants were allowed to withdraw from the study at any time for the following conditions: *1)* withdrawal of consent, *2)* noncompliance, characterized by repeated failure to adhere to study procedures, or *3)* the occurrence of adverse events that required withdrawal on the advice of the research physician or by the participant’s parents’ own decision. Participants who withdrew were not replaced, and all reasons for withdrawal were systematically recorded.

The trial was designed to detect significant differences in body fat mass at 12 mo between the Nordic diet and the conventional diet group. With a 20% dropout rate, 125 infants per group were needed to detect a difference of 0.24 kg in body fat mass while achieving 80% power at a 0.05 significance level (α). Given that the metabolomics profiling was exploratory, it was included as a secondary outcome, and thus, no separate power calculation for metabolomics was performed.

### Ethical considerations

The OTIS study was reviewed and approved by the Regional Ethnics Review Board at Umeå University (2014-363-31M), Umeå, Sweden, and registered at clinicaltrials.gov under the identifier NCT02634749. Written informed consent was obtained from both parents, or from the legal guardians, of all participating infants.

### Dietary intervention procedures

The study utilized a multicomponent approach, incorporating several interventions to implement the Nordic diet portfolio-based complementary foods. A brief overview of the dietary regimens is summarized in Supplementary Table 1. The food introduced in the Nordic diet group was composed exclusively of Nordic ingredients, consistent with the principles established in the New Nordic Food program by the Nordic Council of Ministers [26]. To ensure implementation of the Nordic diet, infants between 4 and 6 mo were introduced to a 24-d taste-portion introduction regimen with repeated exposure to Nordic fruits, berries, and vegetables. Homemade purees featuring Nordic ingredients were sequentially introduced, beginning with sweeter flavors, such as apple, and progressing through green pea, raspberry, cauliflower, lingonberry/buckthorn, turnip, cranberry, and finally white radish. All ingredients, except for buckthorn berry, are available year-round. When buckthorn berry was unavailable, lingonberry was used as a substitute. Parents were advised to offer 3 daily exposures (5–15 mL per exposure) for 3 consecutive days for each type of puree. Every 3 days, a new fruit or vegetable puree was introduced until all 9 had been sampled, allowing infants to taste all options >24 d, for a total of 72 exposures. The composition and sensory profiling of these recipes, as well as the child’s eating behavior questionnaires were reported previously [24].

From 6 to 18 mo, parents were instructed to prepare homemade, protein-reduced baby foods using specific recipes that utilized Nordic ingredients. Protein-reduced, Nordic ingredient-based baby food products from Semper AB, Sweden, were provided, which included specially formulated, protein-reduced Milk Cereal Drinks (MCDs), porridge, and Baby food products In Glass jars (BIGs). When BIG was served as the main course meal, parents were advised to replace half of the BIG portion with homemade fruits and vegetable puree. Parents in the Nordic diet group received recipes for 28 nutritionally balanced, protein-reduced menus based on Nordic diet principles. Each recipe was kept isocaloric by increasing root vegetable content while maintaining fat content. Additionally, 28 fruit (including berries) and vegetable puree recipes were provided beyond the 8 initial taste-portion recipes, along with 10 family-oriented recipes designed to introduce the entire household to the Nordic diet. Parents were invited to a closed Facebook group offering educational support through videos, images, and notifications to assist with food preparation and to enhance motivation to adhere to the study protocol.

In contrast, parents in the conventional group received no additional instruction beyond the national recommendations regarding complementary feeding and weaning, but did receive the standard MCD, porridge, and BIG without protein modification. These parents were free to use any baby food they found to their liking. In both groups, the cereals (MCD and porridge) were iron-fortified and formulated to meet age-specific nutritional requirements. Both groups were also given baby milk drinks. All products, including MCDs, porridges, BIGs, and baby milk drinks, were supplied free of charge and manufactured by Semper AB, Sweden. All participating families, regardless of group assignment, had access to online resources regarding national recommendations from the Swedish Food Agency, representing the standard of care [24].

### Assessment of dietary intake

To evaluate adherence to and acceptance of the protein-reduced Nordic diet complementary feeding regimen, 5-d food records were collected within 2 wk of the 6, 9, 12, and 18 mo study visits. Parents were instructed to document the amount and timing of all food and beverages their child consumed, including breast milk and any food supplements. Daily energy and macronutrient intake, as well as fruit and vegetable consumption, were calculated using Dietist Net Pro (Kost och Näringsdata AB). This software utilizes the Swedish National Food Administration Food Composition Database (version 01/01/2017) and incorporates nutritional information from food manufacturers, including the study foods provided by Semper AB. Mean daily intake across the 5-day dietary records was calculated for each time point. Although the continuation of breastfeeding was documented, the exact quantity of milk consumed was not measured. Instead, breast milk consumption was estimated based on a qualitative categorization as either a “meal” (102 g) or a “snack” (25 g) [24].

### Blood sample collection for metabolomics analysis

EDTA plasma samples were collected by experienced pediatric research nurses via venipuncture blood samples at age 12 and 18 mo after a minimum 2 h fast. To minimize discomfort, a local anesthetic cream was applied prior to blood collection. Once collected, EDTA plasma tubes were allowed to rest for 1 h, then centrifuged at 3000 × g for 10 min at room temperature. The plasma layer was carefully transferred, immediately frozen, and stored at −20°C during the day before being moved to −80°C by the end of the day for storage until NMR-based metabolomics analysis.

### NMR-based metabolomics of infant plasma

Each EDTA plasma sample was prepared for NMR analysis by filtration using a 3 kDa molecular weight cutoff ultracentrifugal filter (Amicon Ultra-0.5 mL, Millipore). Prior to sample filtration, filters were rinsed 3 times with Milli-Q water at 25°C and centrifuged at 14,000 rcf for 5 min, then dried using Kimwipes. Plasma was filtered at 4°C and 14,000 rcf for 40 min, and the filtrate containing small polar metabolites was retained. To 199 μL of the plasma filtrate, 23 μL of an internal standard [5 mM 3-(trimethylsilyl)-1-propanesulfonic acid-d_6_ (DSS-d_6_) and 0.2% NaN_3_ in 99.8% D_2_O] and 8 μL of 1M phosphate buffer were added. The pH was adjusted to 6.8 ± 0.1 using small amounts of HCl and NaOH. Finally, 180 μL of each sample was transferred to 3 mm NMR tubes (Bruker) and stored at 4°C until data acquisition.

All ^1^H-NMR spectra were acquired at 25°C using the noseypr1d pulse sequence on a Bruker Avance 600MHz spectrometer (Bruker) using the procedure described previously [27]. Targeted metabolomics quantification was performed using Chenomx NMRSuite (version v8.6, Chenomx). Each spectrum was Fourier transformed, then manually phase and baseline corrected in Processor. Metabolite quantification was performed manually in Profiler using the concentration of the internal standard (DSS-d6) as reference. Peak fitting and metabolite assignment were confirmed by at least 2 researchers. Targeted metabolomics by ^1^H-NMR offers high reproducibility and quantitative accuracy (±1% accuracy), with a limit of detection in the low micromolar range [28,29]. Plasma metabolites are expressed as μmol/L (μM).

### Other data sources

Demographic and baseline characteristics [maternal prepregnancy BMI (kg/m^2^), gestational weight gain (kg), infant birth weight (g), exclusive breastfeeding duration before enrollment (month), and any formula use before enrollment], infant anthropometry [body weight (kg), length/height (cm) and BMI (kg/m^2^)], infant body composition [fat mass index (kg/m^2^), fat-free mass (kg)], and clinical chemistries including plasma IGF-1 (ng/mL), insulin (mIU/L), folate (nmol/L), and glucose (mmol/L) were extracted from the parent cohort database and have been reported previously [22].

### Statistical analysis

Statistical analysis was performed using R (version 4.5.0), and plots were generated using ggplot2 and ggpubr packages. Metabolite concentrations were transformed to approximate normality using the generalized log transformation [defined as *log(y + sqrt(y*^*2*^*+ lambda)*], where lambda is 1). Principal component analysis (PCA) was performed using the *prcomp* function on the combined 12- and 18-mo dataset, with each variable centered by subtracting the variable means (*center = TRUE*) and scaled by the standard deviation (*scale = TRUE*). Score plots were stratified by timepoint for visualization. Confidence ellipses representing 95% confidence intervals (CI) based on a t-distribution were added to the PCA score plots. To identify which metabolites were contributing the most to principal component 1, a PCA contribution plot was generated by using *fviz_contrib* function from the *factoextra* package. To evaluate overall group differences in the plasma metabolome, we performed permutational multivariate analysis of variance (PERMANOVA) using a nonparametric implementation by the *adonis* function from the *vegan* package. Canberra distance was chosen as the distance metric, and statistical significance was determined using 999 permutations (*nperm* = 999). The assumption of PERMANOVA on homogeneity of dispersion was assessed using the *betadisper* function from the vegan package, where a non-significant result (*P* > 0.05) indicated that this assumption was satisfied.

Pearson’s correlation coefficient was applied to evaluate the strength of correlations. Group differences in baseline characteristics and dropout analyses were evaluated using Chi-squared tests (categorical variables) or Welch’s t-tests (continuous variables). Differences in dietary, biochemical, and metabolite data between groups were assessed using Mann-Whitney *U*-tests (continuous variables) or exact binomial tests (binary categorical variables). False discovery rate (FDR) from multiple hypothesis tests was controlled by the Benjamini-Hochberg procedure, with the overall level of significance set at FDR-adjusted *P* < 0.05.

#### Covariance-based–SEM

Cumulative intake of carbohydrate, protein, fat, and total energy was estimated using the AUC calculated from the mean of 5-d dietary records, where intake was recorded in g/d for macronutrients or kcal/d for total energy. The AUC was determined using the *auc* function from the *MESS* package using linear interpolation and was only applied to subjects with complete data at all 4 time points (6, 9, 12, and 18 mo).

Two covariance-based (CB)–SEM models were constructed using the *lavaan* package, either with infant weight or infant BMI as endogenous variables, while sharing the same set of exogenous variables: cumulative protein intake (estimated from the AUC of protein intake over time), plasma total BCAAs, plasma IGF-1, plasma insulin-to-glucose ratio, infant sex, infant age, birth weight, exclusive breastfeeding duration prior to study enrollment (in months), any formula use before complementary feeding, maternal prepregnancy BMI, and maternal gestational weight gain.

Because both CB-SEM models were specified to evaluate mechanistic relationship based on cumulative protein intake rather than intervention-group assignment, the randomized group assignment was not included separately in the SEM. The group effect is already represented through the observed variation in cumulative protein intake. Both CB-SEM models included only observed variables, with no latent constructs. No imputation was performed for missing data.

To address the issue of variables measured on different scales, plasma total BCAAs were natural-log-transformed, and cumulative protein intake data were standardized to *z*-scores (*mean = 0, SD = 1*). Similarly, IGF-1 and insulin values were natural-log-transformed to reduce skewness and improve heteroscedasticity to satisfy regression assumptions.

SEM was estimated using robust maximum likelihood estimation (MLR), which yields robust SE and scaled test statistics to adjust for non-normality. Model assumptions were assessed using a combination of linear models and linear mixed-effects models with a random intercept to account for the repeated-measures structure of the data. Residuals were examined using the *simulateResiduals* function from the *DHARMa* package, which generated QQ plots, residual versus predicted scatter plots, and results from the Kolmogorov-Smirnov, dispersion, and outlier tests.

Model fit was evaluated using standard indices, including the robust comparative fit index (CFI), incremental fit index (IFI), goodness-of-fit index (GFI), adjusted goodness-of-fit index (AGFI), normed fit index (NFI), robust Tucker-Lewis index (TLI), robust standardized root mean square residual (SRMR), and robust root mean square error of approximation (RMSEA). Modification indices were used to assess specific paths for the best-fitting model, using a χ^2^ value indicating the probability threshold of < 0.05, indicating significant modifications. An example R workflow illustrating the SEM model specification, fitting, and diagnostic checks is provided in the Supplementary Note.

## Results

### Participants characteristics

In the original OTIS trial, 540 participants were screened, and 250 were randomly assigned to 1 of the 2 dietary groups (Nordic diet group, *n* = 125, or conventional diet group, *n* = 125). The mean enrollment age was 4.5 mo (SD 0.5), with 45% of the infants being female. The 4 study visits were conducted at mean ages of 6.3 mo (SD 0.5), 8.7 mo (SD 0.4), 11.8 mo (SD 0.3), and 17.8 mo (SD 0.4). A flow diagram illustrating participant randomization and group allocation process is presented in Supplementary Figure 1. The two dietary treatment groups were well-balanced across all the evaluated baseline characteristics, showing no significant differences in neonatal demographics, family characteristics, breastfeeding duration, and anthropometry data at enrollment between the groups. Baseline demographic and clinical characteristics of all randomized participants by treatment group are reported in Supplementary Table 2.

By age 18 mo, 44 infants (18%) were lost to follow-up. Attrition rate was significantly higher in the Nordic diet group compared with the conventional diet group (Nordic diet: *n* = 30, 24%; conventional diet: *n* = 14, 12%, χ^2^ *P* = 0.013). Dropout analyses revealed no significant differences in infant enrollment age or anthropometric measures. However, infants who dropped out were less likely to have been exclusively or partially breastfed at enrollment. Additionally, mothers of infants in the dropout group were younger and had higher BMI compared with those who completed the study. Fathers’ education levels differed significantly between dropouts and completers, whereas mothers’ education levels did not (Supplementary Table 3). The primary endpoint of the trial, which includes body composition and blood pressure, is reported elsewhere [22]. Other secondary endpoints, as well as adherence measurements, including changes in blood biochemical measurements, dietary intake, eating behavior, and food acceptance, were previously reported [[22], [23], [24]]. Among those that completed the study, 193 (86%) and 181 (88%) at age 12 and 18 mo had plasma samples for metabolomics analysis, which were included in this analysis.

**FIGURE 1:**
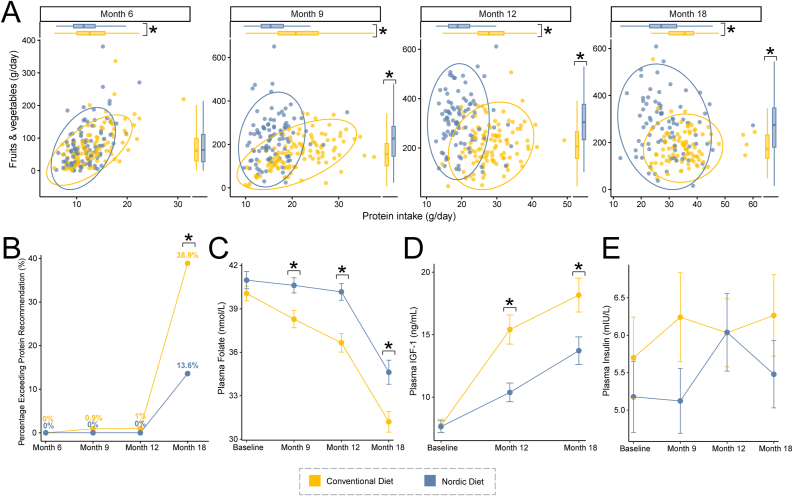
Nordic diet intervention revealed a profound difference in dietary composition and associated adherence markers in infant plasma. (A) Daily intakes of total fruits and vegetables (g/day) and protein (g/day) assessed as mean of 5-day dietary records at age 6, 9, 12, and 18 mo. (B) Percentage of infants exceeding the recommended protein intake threshold (>15% of total energy intake). (C–E) Plasma concentrations of folate, IGF-1, and insulin. Group differences in dietary intakes (A) and biochemical markers (C–E) were assessed cross-sectionally using Mann-Whitney *U*-tests. Group differences in proportions above the protein intake threshold (B) were evaluated using exact binomial tests. *P* values (A–E) were adjusted for multiple comparisons across multiple timepoints using the Benjamini-Hochberg procedure to control the FDR. ∗ Adjusted *P* < 0.05. Abbreviations: IGF-1, insulin-like growth factor 1.

In this intention-to-treat population with metabolomics samples, the proportions of infants receiving any breastfeeding were 71% at 6 months, 39% at 9 months, 20% at 12 months, and 5% at 18 mo. Baseline characteristics of participants included in this secondary analysis are reported in Table 1. At 18 mo, the Nordic diet group exhibited a significantly higher breastfeeding rate compared with the conventional diet group [Nordic diet: 7 (8%), conventional diet: 2 (2%), exact binomial test, FDR-adjusted *P* = 0.009]; no significant differences were observed at earlier time points (6, 9, and 12 mo). Furthermore, 5-d dietary records revealed that the Nordic diet group consistently consumed more fruits and vegetables and less protein (Figure 1A). Interestingly, differences in protein as well as fruit and vegetable consumption between the Nordic and conventional groups were more pronounced at 12 mo than at 18 mo, suggesting decreased adherence to the Nordic diet as time progressed.

**TABLE 1:** Baseline characteristics of study participants included in the secondary analysis.

Characteristic	Nordic Diet (*n* = 98)	Conventional Diet (*n* = 110)
Age at inclusion (mo)	4.4 ± 0.51	4.5 ± 0.51
Sex, *n* (%)		
Female	36 (37)	51 (46)
Male	62 (63)	59 (54)
Birth weight (g)	3609 ± 439	3632 ± 486
Birth length (cm)	51 ± 1.9	51 ± 2.2
Gestational age at birth (wk)	40 ± 1.3	40 ± 1.2
Birth method, *n* (%)		
C-section	18 (18)	18 (16)
Vaginal delivery	80 (82)	92 (84)
Growth parameters at enrollment		
Weight (kg)	7.3 ± 0.85	7.3 ± 0.85
Length (cm)	65 ± 2.3	65 ± 2.4
BMI (kg/m^2^)	17 ± 1.5	17 ± 1.4
HAZ	0.46 ± 0.84	0.5 ± 0.96
WAZ	0.31 ± 0.88	0.35 ± 0.93
WHZ	0.084 ± 0.99	0.11 ± 0.98
BMIZ	0.064 ± 0.97	0.086 ± 0.97
Overall breastfeeding statusat enrollment, *n* (%)		
Exclusive or partial breastfed	97 (99)	109 (99)
Never breastfed	1 (1)	1 (1)
Breastfeeding at enrollment, *n* (%)		
No	22 (22)	27 (25)
Yes	76 (78)	83 (75)
Duration of exclusivebreastfeeding (mo)	4.1 ± 1.4	4.1 ± 1.4
Family characteristics		
Mother’s age	31 ± 4.5	31 ± 4.9
Father’s age	34 ± 4.9	32 ± 5.5
Multiple children, *n* (%)	48 (49)	47 (43)
Maternal prepregnancy BMI	24 ± 4.2	24 ± 3.4
Maternal BMI at childbirth	29 ± 4.6	29 ± 3.7
Maternal BMI at enrollment	25 ± 4.6	25 ± 3.9
Father’s BMI	26 ± 4.4	26 ± 4.2
Mother’s education, *n* (%)		
Below university-level education	28 (29)	34 (31)
University-level education	70 (71)	75 (69)
Father’s education, *n* (%)		
Below university-level education	41 (42)	51 (46)
University-level education	57 (58)	59 (54)

Data represent the analytic population who contributed at least 1 plasma sample for metabolomics analysis at 12 or 18 mo. Data presented as mean ± SD or as numbers (%). Statistical significances were assessed via χ^2^ test (categorical variables) or Welch’s t-test (continuous variables). No significant differences were observed among participants included in the secondary analysis.

Abbreviations: HAZ, height-for-age z-score; WAZ, weight-for-age z-score; WHZ, weight-for-height z-score; BMIZ, BMI z-score.

All infants in both groups met the protein intake recommendations of 1.05 g/d/kg body weight established by the NNR 2023 guidelines [7] at both 12 and 18 months of age. Notably, more infants exceeded the recommended protein intake threshold of 15 E% (percentage of total energy intake) [7,30] at 18 mo compared with earlier visits at 6, 9, and 12 mo. A higher proportion of infants in the conventional diet group consumed protein above this recommendation compared with those in the Nordic diet group (Figure 1B).

The consumption of bitter-tasting leafy greens and many fruits that are naturally rich in folate correlated with significantly higher plasma folate levels in the Nordic diet group compared with the conventional diet group (Figure 1C). Plasma IGF-1 levels, an indicator of elevated protein intake, were significantly lower in this group (Figure 1D). Despite the insulinotropic effect of certain amino acids (eg, leucine, glutamate, and arginine) [31], plasma insulin levels did not differ significantly between groups (Figure 1E). These findings are consistent with data from the full cohort [22], indicating that the metabolomics subset is representative.

### Different complementary feeding regimens reflect infant plasma metabolome

**FIGURE 2:**
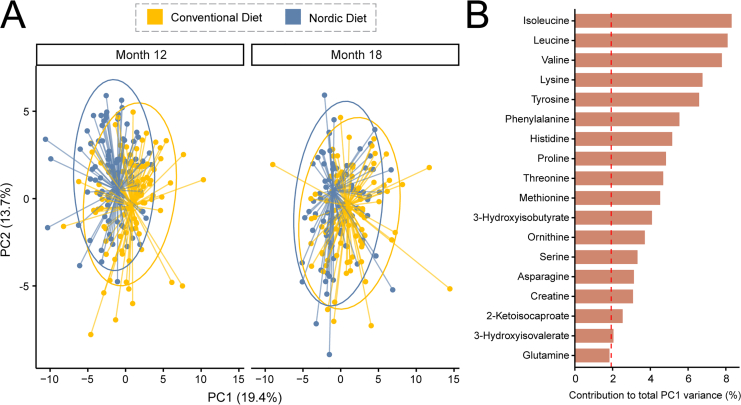
Principal component analysis (PCA) of the plasma metabolome at age 12 and 18 mo. (A) PCA score plots illustrate the compositional difference between groups, with individuals from the same dietary group connected to their respective group centroids. Ellipses indicate 95% confidence intervals based on a t-distribution. PCA was performed on the combined 12- and 18-month dataset and stratified by timepoint for visualization. The variance explained by each principal component reflects the single PCA model. (B) PCA contribution plot identifies which metabolites are most influential to principal component (PC) 1. The red dotted line represents the expected mean contribution of metabolites to PC1; metabolites above this threshold contribute more strongly to the component.

To assess the potential influence of complementary feeding on infant metabolism, targeted ^1^H NMR-based metabolomics was performed on plasma samples collected at age 12 and 18 mo. A total of 52 polar metabolites were quantified. PCA revealed a distinct separation between the 2 dietary groups at both time points, with samples clustering predominantly along the first principal component (PC1) (Figure 2). Amino acids and their derived metabolites were the primary contributors to the total variance captured by PC1. Subsequent distance-based PERMANOVA revealed a significant group difference at 12 mo (R^2^ = 0.022, *P* = 0.008), but not distinct at 18 mo (R^2^ = 0.008, *P* =0.273).

**FIGURE 3:**
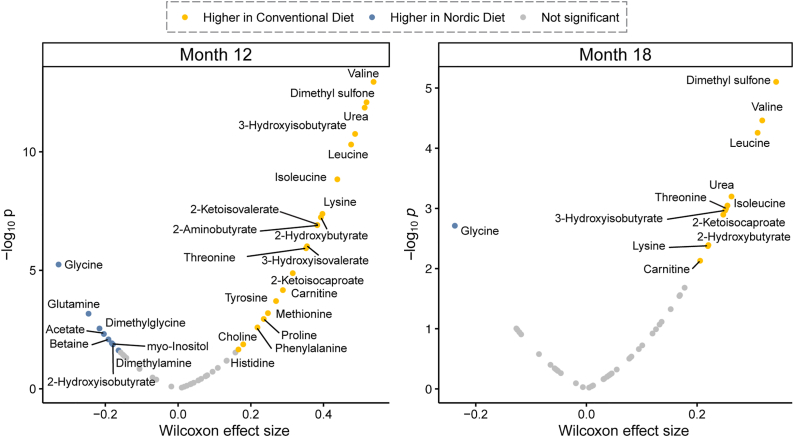
Volcano plots of plasma metabolite differences. Plasma metabolite concentrations between the Nordic and conventional diets at 12 and 18 mo were assessed using the Mann-Whitney *U*-test with Benjamini-Hochberg FDR adjustment, with significance cutoff of *P* < 0.05. Wilcoxon effect sizes were determined with the following thresholds: small (0.1–0.3), moderate (0.3–0.5), or large (>0.5). Abbreviations: FDR, false discovery rate.

These findings were further validated through multiple hypothesis testing using the Mann-Whitney *U*-test with FDR correction (Figure 3). A metabolic signature associated with the Nordic diet was characterized by significantly lower plasma concentrations of markers related to dietary protein intake and metabolism. Specifically, BCAA (leucine, isoleucine, and valine), urea, dimethyl sulfone, lysine, threonine, and BCAA catabolites (2-ketoisovalerate, 2-ketoisocaprate, 2-hydroxybutyrate, and 3-hydroxyisobutyrate) were lower in infants receiving the Nordic diet compared with those on the conventional diet. In addition, circulating carnitine concentrations were significantly higher in the conventional diet group, whereas glycine concentrations were higher among infants on the Nordic diet. Collectively, these results demonstrate that consumption of the Nordic diet results in a distinct plasma metabolomic profile in infants.

### SEM analysis reveals relationship between dietary protein, metabolic markers, and growth outcomes

To test a literature-informed causal framework based on the Early Protein Hypothesis, we applied SEM linking cumulative protein intake with circulating BCAAs (quantified by NMR-based metabolomics) and with previously established IGF-1, insulin, and growth outcomes in the cohort. SEM is a confirmatory approach used to assess prespecified causal pathways and was therefore suitable for evaluating the Early Protein Hypothesis using data from this well-characterized cohort. This sophisticated statistical approach simultaneously estimates all specified paths, allowing us to evaluate the strength of the association between protein intake and growth outcomes while accounting for effects from recent protein intake that are concurrently mediated by BCAAs, IGF-1, insulin, as well as key maternal and neonatal covariates. This approach also allowed us to assess overall model fit using goodness-of-fit indices and to visualize the complex relationships through a path diagram. Because cumulative protein intake represents a post-hoc dietary assessment rather than a randomized assignment, the SEM evaluates these relationships observationally even though the data originate from a randomized trial.

We first defined long-term dietary habits by using dietary intake data collected at 6, 9, 12, and 18 mo to calculate the AUC from 6 to 12 mo and from 6 to 18 mo. These AUC measures represent the cumulative intake from the total diet (breastmilk or infant formula plus complementary foods) during the complementary feeding period. In this cohort, the Nordic diet intervention significantly reduced cumulative protein intake during the complementary feeding period, with a slight increase in cumulative carbohydrate intake, whereas total energy intake remained comparable between groups (Supplementary Figure 2).

Furthermore, congruent with the Early Protein Hypothesis, we created an a priori factor structure showing that high protein intake during the complementary feeding period increases circulating levels of BCAAs, which in turn stimulates the secretion of higher levels of IGF-1 and insulin. We anticipate that this prolonged and cumulative exposure to high protein during this period will accelerate weight gain and increase body fat.

Based on theoretical assumptions, we expected that plasma IGF-1 and insulin concentrations would be strongly influenced by recent dietary protein intake. Therefore, to capture this in our model, we included circulating total BCAAs (sum of leucine, isoleucine, and valine concentrations in plasma), which we confirmed were positively correlated with recent protein intake derived from 5-d dietary records, expressed either as g/d or g/d/kg body weight (Supplementary Figure 3, Pearson R > 0.32, *P* < 0.001). Moreover, strong intercorrelations among the individual BCAAs (leucine, isoleucine, and valine) support the use of total BCAAs as a representative measure instead of using any single BCAA alone (Supplementary Figure 4, Pearson R > 0.93, *P* < 0.001).

Recognizing that infant growth outcomes are multifactorial, we adjusted our analyses for key pre- and postnatal confounders, including maternal gestational weight gain, maternal prepregnancy BMI, infant birth weight, sex, and age, as well as exclusive breastfeeding duration and any infant formula used prior to complementary feeding, both of which were reported at study enrollment (between 4 to 6 mo of age). Infant weight and BMI were chosen as the primary growth outcomes. Although infant length was not modeled as an outcome due to potential measurement inaccuracies, such as variations in positioning the baby and the skill of the measurer, its high correlation with body weight in our dataset suggests that both height-based and weight-based findings can serve as indicators of linear growth (Supplementary Figure 5, Pearson R > 0.68, *P* < 0.001). Body composition data obtained using the D_2_O dilution method, including fat mass and lean mass, were available only for a subset of infants at each visit (72.0% at 12 mo and 49.5% at 18 mo), and therefore were not suitable for modeling as growth outcomes. Nevertheless, validation in this subset of infants showed that fat-free mass index (FMI) was strongly correlated with BMI (Supplementary Figure 6, Pearson R > 0.62, *P* < 0.001), confirming BMI as a good approximation of infant adiposity.

We subsequently employed CB-SEM to examine the relationships among cumulative protein intake, circulating total BCAAs, the insulin-to-glucose ratio, IGF-1, and growth outcomes (infant weight or BMI), while adjusting for the confounders mentioned above. The insulin-to-glucose ratio was used as a proxy measure of insulin concentrations to account for feeding variability in the nonfasted state.

Two SEM models were constructed: one modeling body weight as the growth outcome and the other modeling BMI. Both SEMs examined the relationship between body weight or BMI and cumulative protein intake with concurrent plasma IGF-1 and plasma insulin-to-glucose ratios, while adjusting for infant age and sex as biological confounders. Each model also included paths representing the hypothesized effect of plasma total BCAA on stimulating IGF-1 and insulin-to-glucose ratio, with adjustments for infant age and sex. Although weight-for-age z-scores (WAZ) and BMI z-scores (BMIZ) are commonly reported, we modeled absolute body weight (kg) and BMI (kg/m^2^) because our SEMs already included age and sex as covariates; therefore, further z-score standardization would be redundant.

**FIGURE 4:**
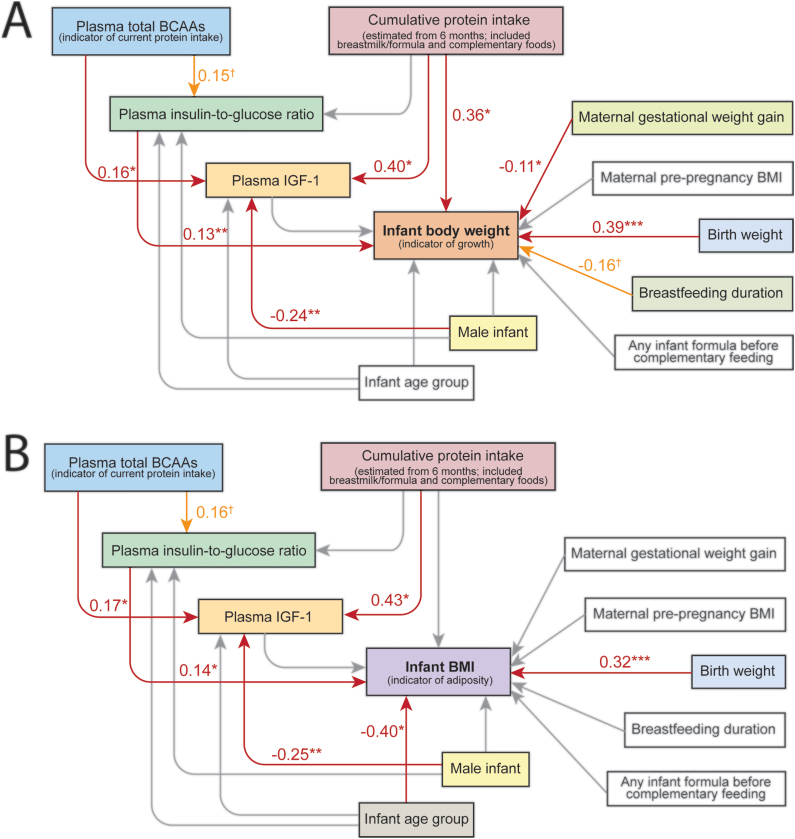
Structural equation model for infant growth and adiposity. This pathway diagram illustrates the structural equation models (SEMs) that analyze how (A) infant weight or (B) infant BMI are influenced by cumulative protein intake from complementary food, plasma total BCAAs, IGF-1, and insulin-to-glucose ratio. These models are adjusted for several confounders, including maternal gestational weight gain, prepregnancy BMI, infant birth weight, sex, and age, as well as exclusive breastfeeding duration and any infant formula use prior to complementary feeding, both of which were reported at study enrollment (between age 4 and 6 mo). Age was modeled as a categorical variable (12 or 18 mo). Cumulative protein intake was estimated using dietary intake data collected at 6, 9, 12, and 18 mo. Specifically, the AUC was calculated for 2 intervals: from 6 to 12 mo and from 6 to 18 mo, representing cumulative protein intake from the total diet during the complementary feeding period. The corresponding AUC (ending at 12 or 18 mo, respectively) and plasma total BCAA, insulin-to-glucose ratio, and IGF-1 from months 12 and 18 were used for these SEM models. The arrows in the SEM path diagram do not imply definitive causal relationships but rather represent hypothesized causal or correlational relationships as modeled in the SEM. Only statistically significant or marginally significant standardized coefficients are labeled on the path diagram: ∗∗∗*P* < 0.001, ∗∗*P* < 0.01, ∗*P* < 0.05, †*P* < 0.1. Comprehensive SEM parameter estimates for both models, including standardized coefficients, t-statistics, 95% confidence intervals, and *P* values for all paths, are presented in Supplementary Table 5. Abbreviations: BCAAs, branched-chain amino acids, IGF-1, insulin-like growth factor 1.

The SEM modeling of infant body weight as the growth outcome demonstrated goodness-of-fit according to established criteria. In contrast, the SEM that employed BMI as the growth outcome showed only moderate fit based on several model fitness indices, suggesting that the weight-based SEM better supports the underlying theoretical framework. The fitting indicators for these models are reported in Supplementary Table 4. The SEM parameter estimates of both SEM models, including standardized coefficients, t-statistics, 95% CI, and *P* values for all paths, are presented in Supplementary Table 5. The corresponding model diagrams are shown in Figure 4.

Maternal gestational weight gain had a significant negative effect on infant weight (β = −0.11), whereas breastfeeding duration showed a marginally negative effect on infant weight (β = −0.16). Infant birth weight (β = 0.39), plasma insulin-to-glucose ratio (β = 0.13), and cumulative protein intake from complementary food and breastmilk or formula combined (β = 0.36) each had a significant positive effect on infant body weight. In contrast, when modeling infant BMI as the outcome, only birth weight (β = 0.32) and plasma insulin-to-glucose ratio (β = 0.14) demonstrated significant positive relationships, whereas increased age was negatively associated with infant BMI (β = −0.40). Notably, cumulative protein intake did not significantly influence infant BMI while controlling for all other variables in the model.

Consistent with established clinical associations, cumulative protein intake during the complementary feeding period was positively associated with plasma IGF-1 concentrations (weight-based SEM: β = 0.40 or BMI-based SEM: β = 0.43) but had no significant relationship with the plasma insulin-to-glucose ratio. Additionally, plasma total BCAAs, an indicator of recent protein intake, were positively associated with plasma IGF-1 concentrations (weight-based SEM: β = 0.16, BMI-based SEM: β = 0.17) and only showed a marginal association with the insulin-to-glucose ratio (weight-based SEM: β = 0.15, *P* = 0.083; BMI-based SEM: β = 0.16, *P* = 0.079). Interestingly, infant sex had a significant influence on plasma IGF-1, with boys exhibiting lower IGF-1 concentrations compared with girls.

This SEM-based analysis demonstrates that cumulative protein intake during the complementary feeding period, after accounting for the impact of recent protein intake-derived impact from plasma IGF-1 and insulin, is significantly associated with increased infant body weight, but not with infant BMI. Consistent with this finding, we observed a weak but statistically significant positive association between cumulative protein intake and infant fat-free mass at 12 mo (Pearson r = 0.20, *P* = 0.02), but not at 18 mo. In contrast, cumulative protein intake showed no significant association at 12 mo, and only a marginally significant association with infant fat mass at 18 mo (Pearson R = 0.19, *P* = 0.06, Supplementary Figure 7A, B). Using linear regression to further evaluate these associations while adjusting for sex and the interaction between sex and cumulative protein intake, only fat-free mass at 12 mo remained significantly correlated with cumulative protein intake (β = 0.56, *P* = 0.038, Supplementary Figure 7C).

## Discussion

The Early Protein Hypothesis proposes that early-life protein intake exceeding physiological needs leads to higher circulating BCAAs and IGF-1 than a typical intake would produce. This elevation in turn activates the mechanistic target of rapamycin complex 1 (mTORC1) pathway, an anabolic signal promoting accelerated weight gain during infancy [32]. Epidemiological data from the European Childhood Obesity Project (EU CHOP) trial [[14], [15], [16]], the Dortmund Nutritional and Longitudinally Designed (DONALD) study [33,34], among others [13,35], have demonstrated that high protein intake during infancy is associated with more rapid growth during infancy and an increased risk of overweight or obesity later in childhood. Rapid weight gain (a change in WAZ > 0.67) during the first 2 y of life is itself an independent predictor of subsequent obesity across the life course [36,37]. These observations have motivated the development of better complementary foods. One such diet is the Nordic diet with a reduced-protein level.

In our randomized controlled trial, infants were assigned to receive either a protein-reduced, fruit and vegetable-enriched Nordic diet or a conventional complementary diet from age 4–6 mo to 18 mo. Although the Nordic diet group exhibited reduced circulating IGF-1 and BCAA concentrations, there were no significant differences between the groups in the primary end point of the study on body composition, weight gain, length/height gain, and BMI at 12 and 18 mo [22]. Dietary intake data also revealed more pronounced differences at 12 mo compared with 18 mo. These findings may be partly explained by the fact that approximately one-third of infants in the Nordic diet group started attending daycare or preschool starting from age 1 y, which led to increased protein and reduced fruit and vegetable intake outside of the home. Consequently, dietary patterns of infants from the Nordic diet group became more similar to the conventional group, which represents the typical complementary feeding practices in Sweden.

In this secondary analysis, we first demonstrated the impact of a reduced-protein Nordic diet on infant plasma. We observed that many circulating amino acids and their derived metabolites were significantly lower in the Nordic diet group compared with the conventional group, predominantly at 12 mo. Consistent with the dietary difference, circulating carnitine concentrations, a biomarker of red meat consumption [38], and blood urea, a nitrogenous waste product of protein metabolism, were significantly lower in infants consuming the Nordic diet compared with those in the conventional diet group at 12 mo. Additionally, elevated dimethylamine concentrations in the Nordic diet group may reflect higher fish and seafood intake [38]. Circulating betaine levels were also higher in infants following the Nordic diet at 12 mo, in line with a previous finding in Swedish adults adhering to a Nordic diet [39]. This increase likely reflects the inclusion of Nordic food ingredients that are high in betaine, such as beets, spinach, whole grains, and shrimp [40].

Circulating glycine levels were higher in the Nordic diet group, which is consistent with another randomized controlled trial in obese adults that reported increased plasma glycine concentrations in individuals consuming a New Nordic diet compared with those consuming a typical Danish diet [41]. Elevated glycine has also been identified as a metabolic signature in Finnish children with a high healthy eating index and is associated with lower consumption of red meat [42]. Glycine is recognized for its anti-inflammatory and antioxidant properties, due to its function in inhibiting NF-κB (Nuclear factor-κB) signaling and its role as a precursor in glutathione synthesis [43,44]. Additionally, concurrent low glycine and elevated BCAA have been shown to be markers of insulin resistance and type 2 diabetes [45,46] and are causally linked to a higher risk of developing hypertension and coronary heart disease [47]. Because elevations of BCAA and their catabolites can mechanistically contribute to mitochondrial stress-associated insulin resistance [48], glycine becomes crucial for counteracting this metabolic burden through its roles in clearance of accumulating short-chain acyl-CoA [49,50] and glutathione production [51]. For these reasons, glycine has also been viewed as conditionally essential in obesity, insulin resistance, and diabetes [52]. Although the Developmental Origins of Health and Disease (DOHaD) framework has proposed that early metabolic exposures may program susceptibility to future metabolic disorders, the long-term implications of such diet-driven metabolic phenotypes in infancy, including alterations in circulating glycine and BCAA, warrant long-term follow-up.

Although randomized controlled trials remain the gold standard for assessing intervention effects, high variability in dietary adherence at 18 mo may have contributed to the lack of direct group differences in infant weight and BMI observed in the original study. Nonetheless, by leveraging detailed dietary, growth, and biochemical data, this secondary analysis integrating plasma metabolomic results with SEM provides further understanding of the metabolic impact of early-life protein intake. According to multiple model fit indices, the SEM for infant body weight demonstrated good fit, whereas the SEM for infant BMI had only moderate fit. The moderate fit for BMI suggests that additional, unmeasured, or residual confounding factors may influence BMI outcomes.

After controlling for various neonatal and maternal confounders, we demonstrated a significant association between cumulative protein intake during complementary feeding and increased infant body weight. However, we did not detect a clear relationship between protein intake and infant BMI up to 18 mo, likely due to the short follow-up period and inherent variability in infant BMI around this age, which typically peaks at ∼12 mo [53]. This natural fluctuation in BMI during infancy may mask the effect of cumulative protein intake occurring between 6 and 18 mo. Because of rapid developmental changes in fat and lean mass, BMI during infancy is inherently unstable and is not suitable for classifying obesity at this age group; in contrast, weight-based indicators are well-established predictors of later obesity risk [36,37,54]. Our interpretation aligns with findings from the previous studies, which suggest that high protein intake exceeding physiological needs during infancy leads to accelerated growth at the time of exposure [15], whereas the effects on adiposity may become apparent later in childhood [13,16,55].

### Strengths and limitations

This study is a randomized controlled trial conducted on a relatively large sample of healthy infants (*n* = 250), providing comprehensive analytical plasma metabolomics profiling (52 metabolites) along with detailed dietary, anthropometric, and biochemical measurements. This rich dataset has enabled a thorough exploratory analysis examining the metabolic effects of complementary feeding regimens during infancy. Although adherence to the assigned Nordic diet varied across families, the intervention created a measurable group-level shift in feeding behavior. In contrast to observational cohorts, where short-term dietary recalls often reflect acute and fluctuating intake, this cohort’s randomized structure provided more stable and interpretable dietary data and metabolic profiles that offer more insight into habitual dietary patterns.

The generalizability of our findings may be limited, as the study included only healthy, well-nourished infants born in Sweden. Thus, the applicability of this complementary feeding intervention to infants in low- and middle-income settings or in other ethnic groups remains unclear. Additionally, potential adherence challenges at 18 mo may have attenuated observed dietary effects between groups. Although BMI is generally considered a suboptimal indicator of body fat mass, our data confirmed a strong correlation between BMI and FMI measured via the deuterium dilution method, supporting its use as a reliable proxy for adiposity assessment in larger infant populations. Additionally, the dietary data did not specify the source of protein, for example, plant- or animal-based. Dropout analyses suggested possible attrition bias, as maternal BMI, breastfeeding status, and paternal education levels differed between participants who completed the study and those who withdrew from the study. These associations cannot be interpreted as causal.

Dietary protein is rarely consumed in isolation and is often accompanied by other dietary components. For example, infants in the Nordic diet group exhibited reduced cumulative protein intake along with increased cumulative carbohydrate intake, likely due to their higher fruit and vegetable consumption. Diets enriched with fruits and vegetables are widely recognized for health benefits, partly due to their high fiber content, which enhances satiety and positively modulates gut microbiota, thereby supporting a healthier metabolic phenotype. However, our SEM model only evaluated a protein-focused hypothesis and did not account for complex interactions between protein and other macronutrients/food components, or their collective impact on gut microbiota.

In conclusion, with childhood obesity rates continuing to rise globally, early metabolic programming has become an important research focus. In this study, we demonstrated that complementary feeding patterns during infancy led to unique plasma metabolomic signatures. Circulating BCAAs and their catabolites concentrations related to dietary protein intake were significantly and consistently lower in infants following the protein-reduced Nordic diet. Beyond their role as markers of protein consumption, circulating BCAAs can exert anabolic signaling effects influencing growth trajectories. Using SEM, we provided evidence that in well-nourished, healthy infants from a resource-rich setting, whose protein intake tended to be substantially higher than recommended, reducing protein intake during complementary feeding attenuates higher infant weight gain, an established early-life predictor of later obesity risk. Our findings also illustrate how metabolite profiles, particularly BCAAs, can help elucidate the pathways linking diet to early growth. Further studies with longer follow-up periods are warranted to fully understand the impacts of early dietary interventions on adiposity and long-term metabolic health.

## Author contributions

The authors’ responsibilities were as follows – TL, OH, BL, UJ, and CMS conceptualized the study. TL and UL oversaw the original study, coordinated its implementation, and contributed substantially to the clinical data curation. XH performed statistical analyses, created visualizations, and wrote the final manuscript. ZZ drafted the original manuscript. ZZ and XH generated the metabolomics data. DJT provided statistical consultation. CMS holds primary responsibility for the manuscript’s final content. ZZ, UJ, DJT, OH, BL, TL, and CMS critically reviewed and revised the manuscript. All authors have read and approved the final manuscript for publication.

## Data availability

Data described in the manuscript and analytical code are available on reasonable request pending application and approval.

## Funding

This study was supported by grants from the Regional Agreement between Umeå University and Västerbotten County Council in Cooperation in the field of Medicine, Odontology and Health (ALF, grants VLL-644531, VLL-488901, VLL- 677921, VLL-761381) (TL), Oskar Foundation (TL), and Semper AB, Sweden (TL and CMS). The funding sources had no involvement or restrictions regarding publication. CMS was also supported by the U.S. Department of Agriculture - National Institute of Food and Agriculture (USDA-NIFA) Hatch project 1021411 and would like to acknowledge support from the Kinsella Endowed Chair in Food, Nutrition, and Health.

## Conflict of interest

TL, OH, and BL have received research support from Semper AB and Hero unrelated to this study. UJ was a doctoral student at the Umeå University Industrial Doctoral School for Research and Innovation, with Semper AB as the industrial sponsor. The funders had no role in study design, data collection, analysis, decision to publish, or manuscript preparation. All other authors declare no conflicts of interest.
