# Supplementary material for: Metabolic impact of reduced-protein Nordic diet-based complementary feeding: a secondary analysis of a randomized controlled study
Source: Am J Clin Nutr. 2026 Feb 16;123(4):101238. doi: 10.1016/j.ajcnut.2026.101238 (PMC13084568; doi:10.1016/j.ajcnut.2026.101238)
Supplement: multimedia component 1 [file mmc1.pdf]

Supplementary Information

Metabolic impact of reduced-protein Nordic diet-based complementary feeding: a secondary analysis of a randomized controlled study

Xuan He[1,2], Zhichao Zhang[1], Ulrica Johansson[3], Daniel J. Tancredi[4], Olle Hernell[3], Bo Lönnerdal[1], Torbjörn Lind[3], Carolyn M. Slupsky[1,2]

[1] Department of Nutrition, University of California-Davis, Davis, CA, United States

[2] Department of Food Science and Technology, University of California-Davis, Davis, CA, United States

[3] Department of Clinical Sciences, Pediatrics, Umeå University, Sweden

[4] Department of Pediatrics, School of Medicine, University of California-Davis, Davis, CA, United States

Table of Contents

Supplementary Figures

- **Supplementary Figure 1.** Participant flow diagram of OTIS, a randomized controlled trial on the effects of protein-reduced, Nordic complementary diet.
- **Supplementary Figure 2.** Group difference in accumulated protein intake.
- **Supplementary Figure 3.** Correlation between recent protein intake and plasma BCAA concentrations.
- **Supplementary Figure 4.** Inter-correlation among individual plasma branched-chain amino acids.
- **Supplementary Figure 5.** Correlation between infant weight and length/height measurements.
- **Supplementary Figure 6.** Correlation between infant fat mass index (FMI) and body mass index (BMI).
- **Supplementary Figure 7.** Correlation between accumulated protein intake (standardized score) and infant fat-free mass and fat mass.

Supplementary Tables

- **Supplementary Table 1.** Dietary regimens in Nordic and Conventional Diets.
- **Supplementary Table 2.** Comparison of baseline characteristics between completed participants and dropouts in the OTIS study
- **Supplementary Table 3.** Comparison of baseline characteristics between completed participants and dropouts in the OTIS study
- **Supplementary Table 4.** Model fitness indices for the structural equation model
- **Supplementary Table 5.** Standard parameter estimates of the structural equation model

Supplementary Note

- **Supplementary Note.** Example R workflow for the structural equation model (SEM)

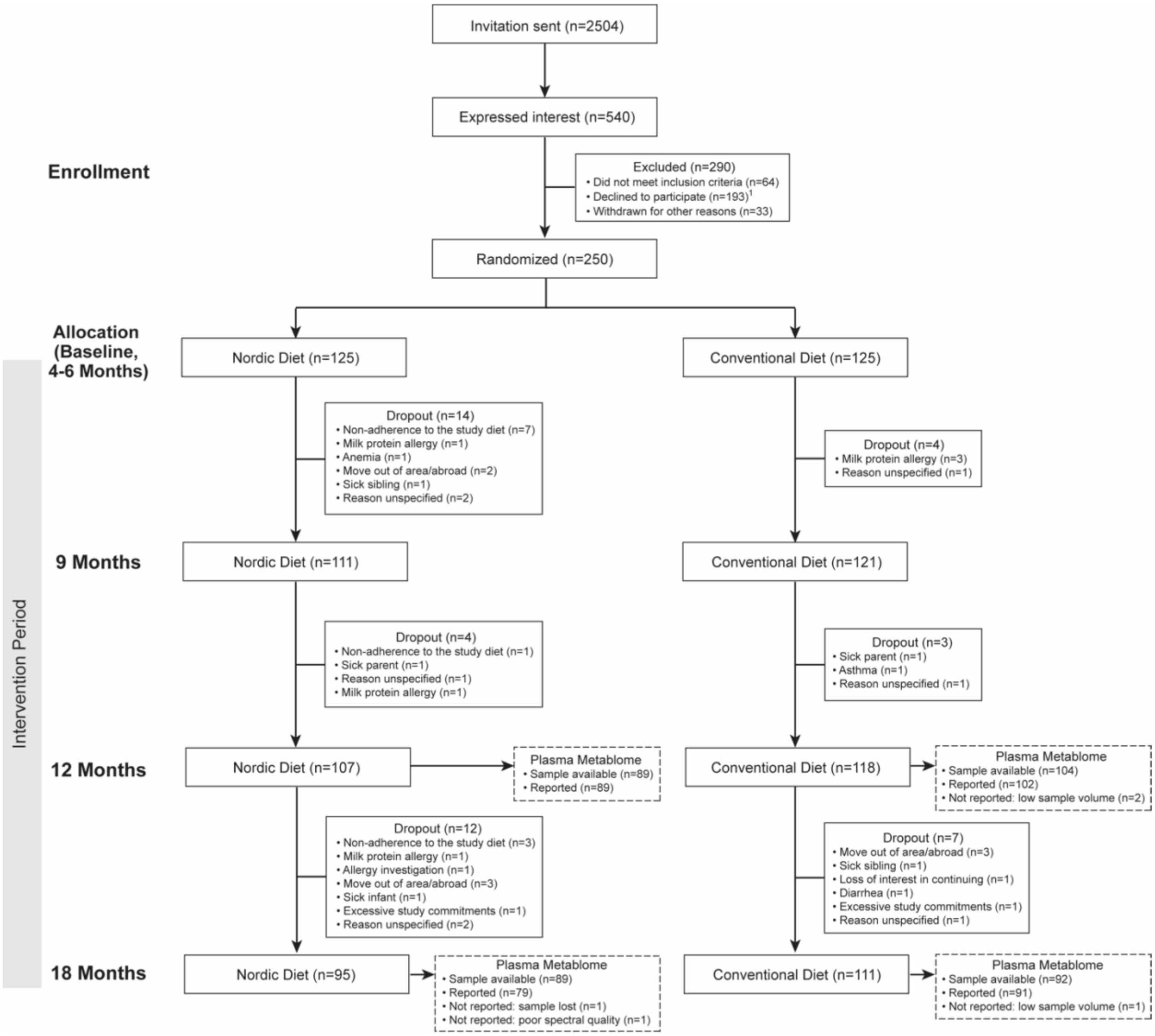

Supplementary Figure 1. Participant flow diagram of OTIS, a randomized controlled trial on the effects of protein-reduced, Nordic complementary diet. [1]Reasons for declining participation at the initial screening stage includes: 1) concerns about the high level of commitment required, 2) the long duration of the study period, 3) unwillingness to adhere to the study diet, and 4) anticipated early start of daycare for the child. OTIS is a Swedish acronym for Optimized Complementary Feeding Study.

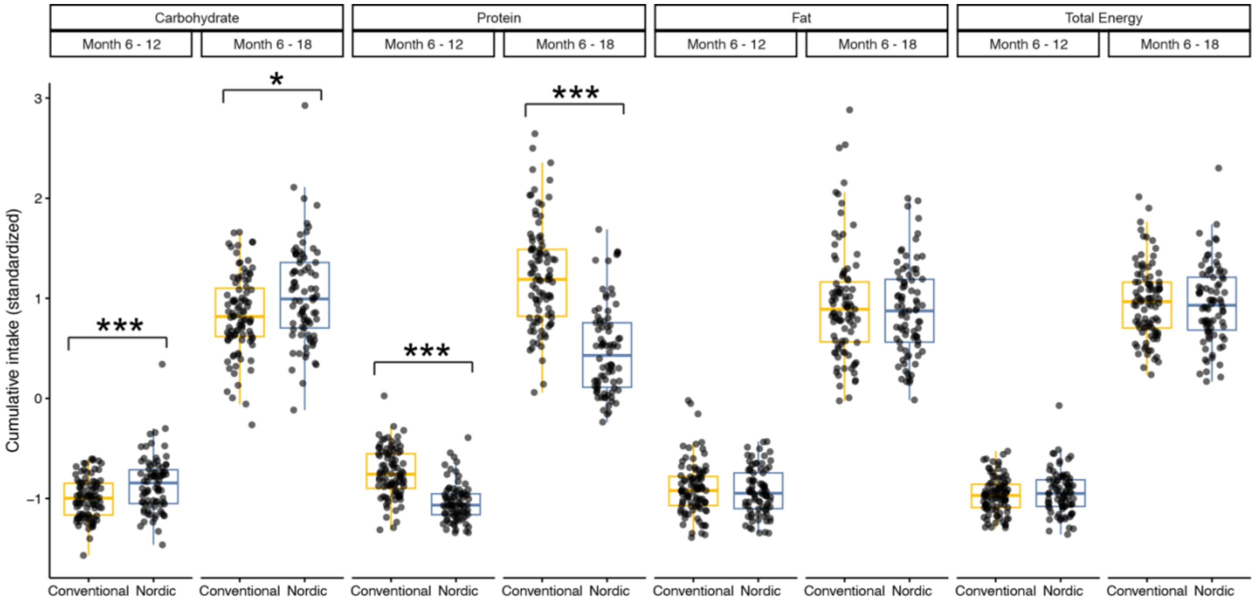

Supplementary Figure 2. Group difference in cumulative intake of carbohydrate, protein, fat and total energy. Cumulative intake was calculated as standardized area under the curve from 6 to 12 months and from 6 to 18 months. Only participants with dietary records available at all four time points (6, 9, 12 and 18 moths) were included in this analysis. Group differences were evaluated via Mann-Whitney U test. *** p<0.001

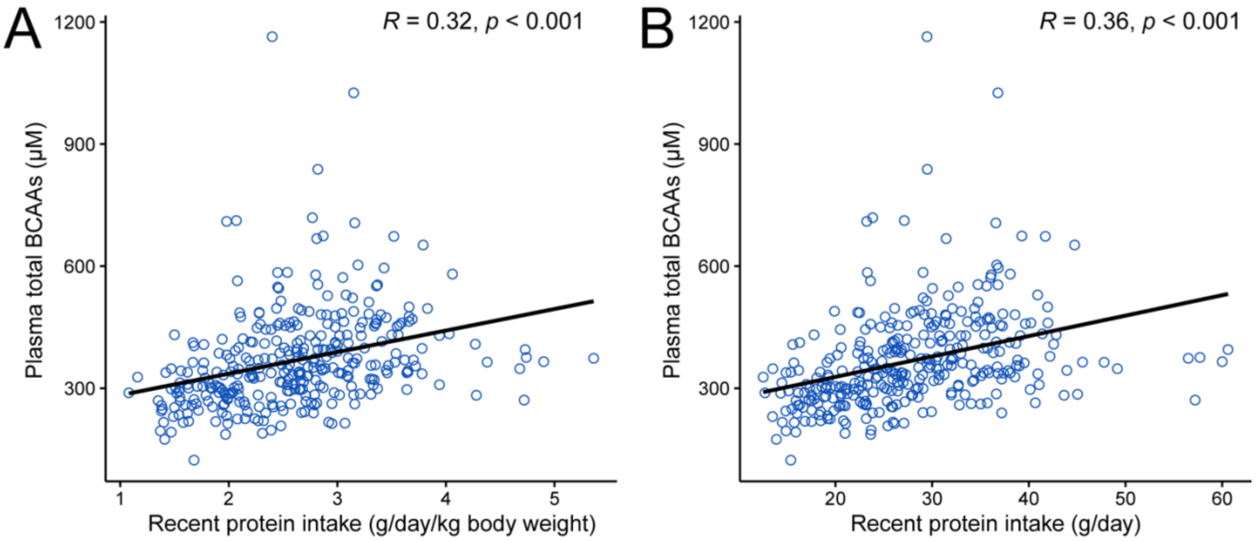

Supplementary Figure 3. Correlation between recent protein intake and plasma BCAA concentrations. Plasma total branched-chain amino acid (BCAA) concentration was determined by summation of leucine, isoleucine, and valine concentrations (in μM). Recent protein intake calculated as the average over a 3-day period, expressed as either (A) g/day/kg body weight or (B) g/day. Pearson's correlation was applied to evaluate the relationship between plasma total BCAA and recent protein intake. Analyses incorporated data from Month 12 (n=190) and Month 18 (n=170).

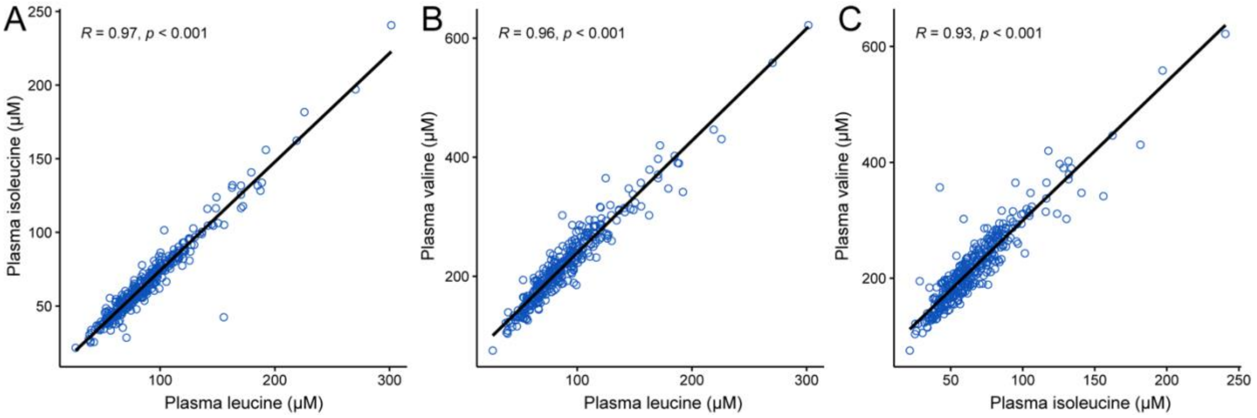

Supplementary Figure 4. Inter-correlation among individual plasma branched-chain amino acids. Pearson's correlation was applied to evaluate the relationships between leucine, isoleucine and valine. Data from Month 12 (n=190) and Month 18 (n=170) were used in these analyses.

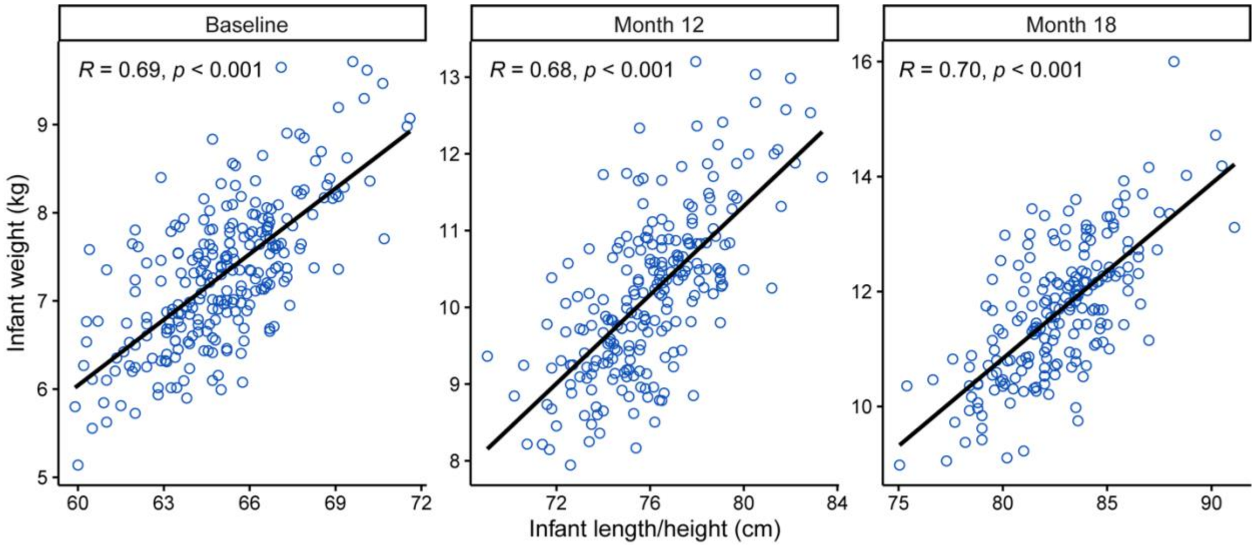

Supplementary Figure 5. Correlation between infant weight and length/height measurements. Pearson's correlation was used to evaluate the relationship between height and weight. Data for these analyses was obtained from Baseline (n=250), Month 12 (n=222) and Month 18 (n=201) measurements.

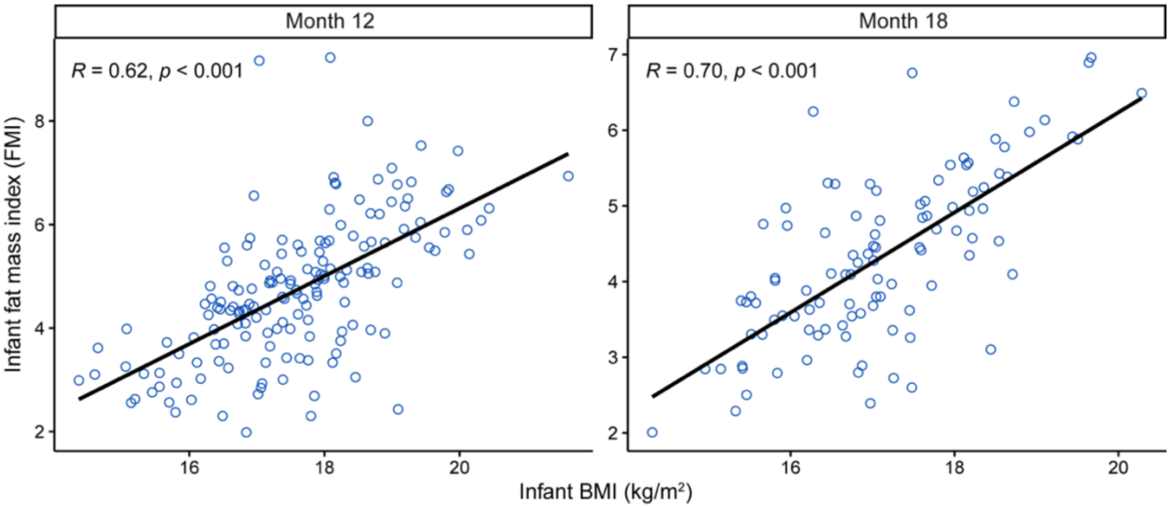

Supplementary Figure 6. Correlation between infant fat mass index (FMI) and body mass index (BMI). Pearson's correlation was used to evaluate the relationship. Data for these analyses were obtained at Month 12 (n=161) and Month 18 (n=102). FMI is determined as fat mass / length^2.

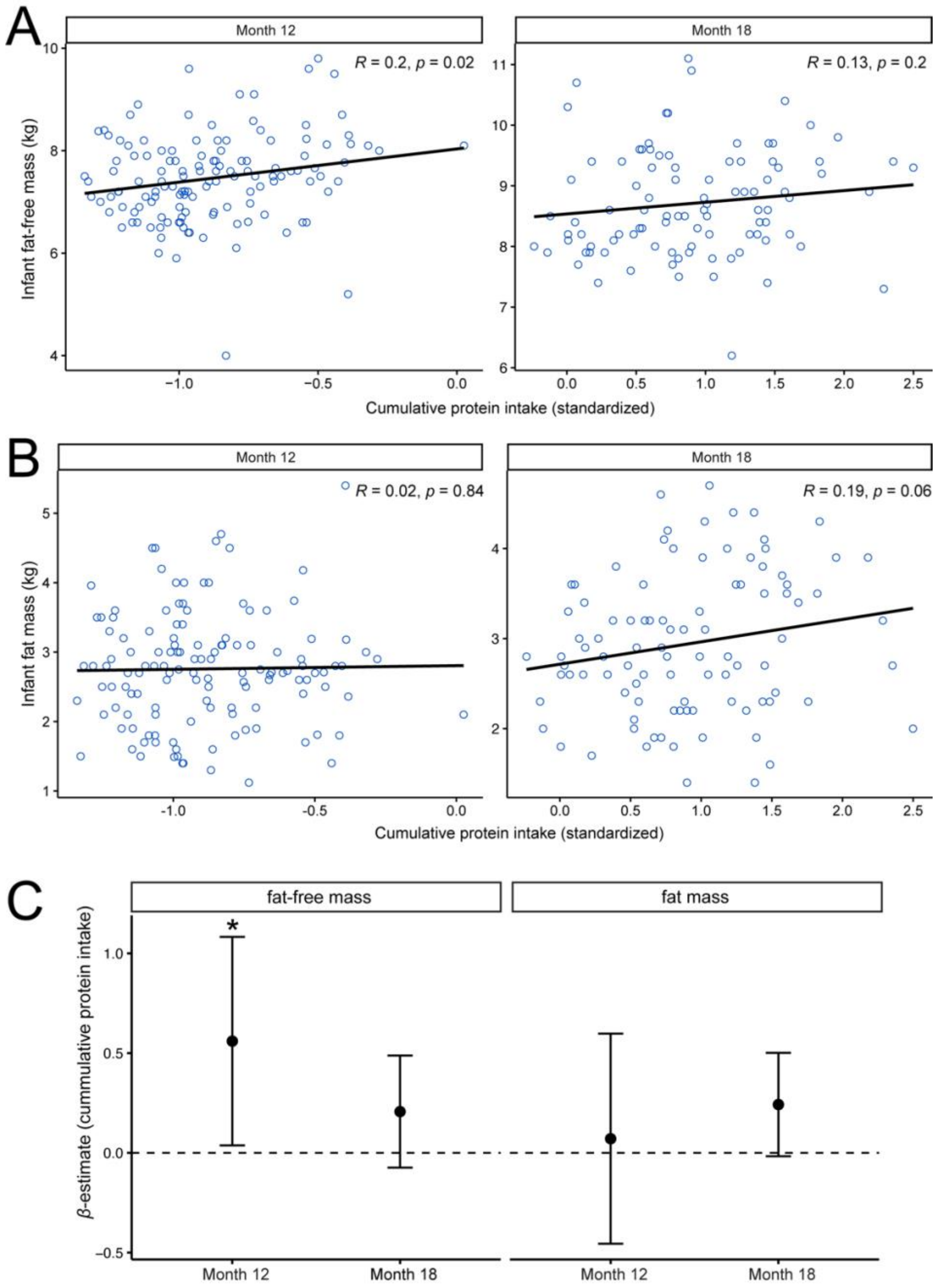

Supplementary Figure 7. Correlation between cumulative protein intake (standardized score) and infant fat-free mass and fat mass. Pearson's correlation analyses were conducted to evaluate relationships between cumulative protein intake scores and (A) infant fat-free mass and (B) infant fat mass. (C) Sex-adjusted association between standardized cumulative protein intake and body-composition outcomes. β-estimates with 95% confidence intervals from linear regression models are shown. All linear regressions were controlled for sex and sex to the interaction between sex and cumulative protein intake. The dashed horizontal line represents the null hypothesis (β = 0). * indicates statistically significant association with sex adjustment (p<0.05). Cumulative protein intake was estimated using dietary intake data collected at 6, 9, 12, and 18 months. Specifically, the area under the curve (AUC) was calculated for two intervals: from 6 to 12 months and from 6 to 18 months, representing cumulative protein intake from the total diet during the complementary feeding period. For cross-sectional analyses at 12 and 18 months, the corresponding AUC (ending at 12 or 18 months, respectively) was used. Data were obtained at Month 12 (n = 131) and Month 18 (n = 96).

Supplementary Table 1. Dietary regimens in Nordic and Conventional Diets[1].

Age	Dietary Actions	Nordic Diet	Conventional Diet
Birth – study enrollment (4-6 months)	Exclusively feeding of human milk or infant formula		
4-6 months	Introduction of complementary food alongside human milk or formula feeding[2]	Provided homemade Nordic baby food recipes and instruction on 24-days taste portion introduction that sequentially introduce a new fruit or vegetable every 3 days. **(Mandatory)**	No additional instruction.
6-18 months	Initial baby cereal-based complementary food introduction.	✓ Specially designed food provided.<br><br>Protein-reduced MCD, porridge and baby milk drink **(Recommended)**	✓ Specially designed food provided.<br><br>Regular MCD, without any protein modification, porridge and baby milk drink **(Optional)**
	Use of commercial baby food products	✓ Specially designed food provided.<br><br>Protein-reduced BIG made with Nordic diet ingredients. Advise to serve only half a portion of the BIG, substituting the remaining half with fruit and vegetable puree. (**Recommend**)	✓ Specially designed food provided.<br><br>Regular BIG, without any protein modification. No additional instruction on whether it should be consumed together with other food. **(Optional)**<br><br>Free to use any baby food they found to their liking
	Preparation of homemade meals[7]	Provided 28 recipes for nutritionally balanced main course meals and an additional 28 recipes for fruit and vegetable purees, all based on the Nordic diet concept but reduced in protein. Eight of these recipes were part of the taste portion schedule between 4 and 6 months. **(Highly Recommended)**<br>Provided 10 family recipes designed to introduce Nordic food to the entire household.	No additional instruction.

[1] Protein content of study products:

- **Protein-reduced MCD:** 1.2 - 1.5 g protein per 100 mL (ready-to-consume)
- **Regular MCD:** 1.9 - 2.2 g protein per 100 mL (ready-to-consume)
- **Protein-reduced porridge:** 2.3 - 3.0 g protein per 30 g serving
- **Regular porridge:** 3.8 - 4.8 g protein per 30 g serving
- **Baby milk drink:** 1.3 - 1.4 g protein per 100 mL (ready-to-consume; not protein-reduced)
- **Protein-reduced BIG:** 1.8 - 2.9 g protein per 100 g puree
- **Regular BIG:** 2.4 - 4.0 g protein per 100 g puree

Fruits and vegetables were supplied to participating families free of charge. Families in the Nordic diet group specifically received locally sourced produce classified as Nordic ingredients, whereas families in the conventional group received commonly available fruits and vegetables in stores year-round.

[2] Swedish National Food Agency recommends parents introduce taste portions alongside breastfeeding or formula feeding if the child is interested in other foods.

Abbreviations: MCD, Milk cereal drink; BIG, Baby Food in Glass Jar.

✓ indicates that specially designed food products were provided by Semper AB, free of charge.

Supplementary Table 2. Baseline characteristics of study participants from the OTIS cohort.

Characteristic	Nordic Diet (n=125)	Conventional Diet (n=125)
Age at inclusion (month)	4.5 ± 0.49	4.5 ± 0.50
Sex		
- Female	52 (42%)	56 (45%)
- Male	73 (58%)	69 (55%)
Birth weight (g)	3626 ± 431	3635 ± 474
Birth length (cm)	51 ± 1.9	51 ± 2.1
Gestational age at birth (wk)	40 ± 1.3	40 ± 1.3
Birth method		
- C-section	23 (19%)	21 (17%)
- Vaginal delivery	101 (81%)	104 (83%)
Growth parameters at enrollment		
Weight (kg)	7.3 ± 0.81	7.3 ± 0.82
Length (cm)	65 ± 2.2	65 ± 2.4
BMI (kg/m^2)	17 ± 1.4	17 ± 1.4
HAZ	0.45 ± 0.81	0.50 ± 0.93
WAZ	0.31 ± 0.83	0.36 ± 0.89
WHZ	0.091 ± 0.95	0.14 ± 0.94
BMIZ	0.069 ± 0.93	0.11 ± 0.93
Overall breastfeeding status at enrollment		
- Exclusive or partial breastfed	123 (98%)	123 (98%)
- Never breastfed	2 (2%)	2 (2%)
Breastfeeding at enrollment		
- No	30 (24%)	31 (25%)
- Yes	95 (76%)	94 (75%)
Duration of exclusive breastfeeding (month)	4.1 ± 1.5	4.2 ± 1.4
Family characteristics		
Mother's age	31 ± 4.6	31 ± 4.9
Father's age	34 ± 5	32 ± 5.4
Multiple children	60 (49%)	56 (45%)
Maternal pre-pregnancy BMI	24 ± 4.5	24 ± 3.5
Maternal BMI at childbirth	30 ± 4.9	29 ± 3.7
Maternal BMI at enrollment	25 ± 4.8	25 ± 4.0
Father's BMI	26 ± 4.2	26 ± 4.2
Mother's education		
- Below university-level education	36 (30%)	40 (32%)
- University-level education	86 (70%)	84 (68%)
Father's education		
- Below university-level education	54 (44%)	58 (46%)
- University-level education	68 (56%)	67 (54%)

Data presented as mean ± SD or as numbers (%). Statistical significances were assessed via Chi-squared test (categorical variables) or Welch's t-test (continuous variables). No significant differences were observed in the baseline characteristics between the two dietary groups. **Abbreviations**: HAZ, z-score for height-for-age; WAZ, z-score for weight-for-age; WHZ, z-score for weight-for-height; BMIZ, z-score for body mass index, OTIS is a Swedish acronym for Optimized Complementary Feeding Study.

Supplementary Table 3. Comparison of baseline characteristics between completed participants and dropouts in the OTIS study

**Characteristic**	**Completer (n=206)**	**Dropout (n=44)**	**Test**
Age at inclusion (month)	4.4 ± 0.47	4.6 ± 0.6	F=2.277
Sex			χ2=2.268
- Female	84 (41%)	24 (55%)	
- Male	122 (59%)	20 (45%)	
Birth weight (g)	3621 ± 465	3676 ± 390	F=0.548
Birth length (cm)	51 ± 2.0	51 ± 1.8	F=0.013
Gestational age at birth (wk)	40 ± 1.3	39 ± 1.4	F=2.551
Birth method			χ2=0
- C-section	36 (17%)	8 (19%)	
- Vaginal delivery	170 (83%)	35 (81%)	
Growth parameters at enrollment			
Weight (kg)	7.3 ± 0.82	7.2 ± 0.80	F=0.353
Length (cm)	65 ± 2.3	65 ± 2.0	F=0.673
BMI (kg/m^2)	17 ± 1.4	17 ± 1.4	F=0.017
HAZ	0.5 ± 0.89	0.37 ± 0.78	F=0.796
WAZ	0.35 ± 0.86	0.28 ± 0.86	F=0.221
WHZ	0.11 ± 0.95	0.12 ± 0.94	F=0.008
BMIZ	0.087 ± 0.94	0.095 ± 0.92	F=0.003
Overall breastfeeding status at enrollment			χ^2=5.651*
- Exclusive or partial breastfed	205 (100%)	41 (93%)	
- Never breastfed	1 (0%)	3 (7%)	
Breastfeeding at enrollment			χ^2=1.142
- No	47 (23%)	14 (32%)	
- Yes	159 (77%)	30 (68%)	
Duration of exclusive breastfeeding (month)	4.1 ± 1.4	4 ± 1.5	F=0.141
Family characteristics			
Mother's age	31 ± 4.7	30 ± 4.8	F=3.981*
Father's age	33 ± 5.2	32 ± 5.5	F=1.246
Multiple children	91 (44%)	25 (61%)	X^2=3.23
Maternal pre-pregnancy BMI	24 ± 3.5	26 ± 5.8	F=5.961*
Maternal BMI at childbirth	29 ± 3.9	31 ± 5.8	F=5.269*
Maternal BMI at enrollment	25 ± 4.0	27 ± 5.9	F=4.892*
Father's BMI	26 ± 4.3	26 ± 3.7	F=0.051
Mother's education			χ^2=1.101
- Below university-level education	60 (29%)	16 (39%)	
- University-level education	145 (71%)	25 (61%)	
Father's education			χ^2=4.12*
- Below university-level education	87 (42%)	25 (61%)	
- University-level education	119 (58%)	16 (39%)	

Data presented as mean ± SD or as numbers (%). Statistical significances were assessed via Chi-squared test (categorical variables) or Welch's t-test (continuous variables). No significant differences were observed in the baseline characteristics between the two dietary groups. * P<0.05. **Abbreviations**: HAZ, height-for-age z-score; WAZ, weight-for-age z-score; WHZ, weight-for-height z-score; BMIZ, body mass index z-score, OTIS is a Swedish acronym for Optimized Complementary Feeding Study.

Supplementary Table 4. Model fitness indices for the structural equation model

	npar	χ^2 fit statistics (scaled)	χ^2 df	χ^2 P value	CFI (Robust)	IFI	GFI	AGFI	NFI	TLI (Robust)	SRMR (Robust)	RMSEA (Robust)
Model for Infant body weight outcomes	24	19.247	12	0.083	0.981	0.981	0.999	0.994	0.955	0.953	0.034	0.061
Model for Infant body BMI outcomes	24	20.603	12	0.056	0.850	0.879	1.000	0.996	0.769	0.625	0.036	0.068
Recommended threshold				>0.05	>0.90	>0.90	>0.90	>0.95	>0.95	>0.95	<0.08	<0.05 good; <0.08 acceptable

Abbreviations: npar, number of parameters; df, degree of freedom; CFI, comparative fit index; IFI, incremental fit index; GFI, goodness-of-fit index; AGFI, adjusted goodness-of-fit index; NFI, normed fit index; TLI, Tucker-Lewis index; SRMR: Standardized root mean square residual; RMSEA: root mean square error of approximation.

Supplementary Table 5. Standard parameter estimates of the structural equation model

Hypothesis	Standardized path coefficient	T statistic	95% CIs (Lower-Upper Bounds)	P value	Supported
Model for Infant body weight outcome					
Cumulative protein intake → Infant weight	0.358	2.055	(0.023, 0.988)	0.040*	Yes
Infant age group → Infant weight	0.200	1.350	(-0.251, 1.365)	0.177	
Infant sex (male) → Infant weight	0.033	0.460	(-0.315, 0.509)	0.645	
Infant birth weight → Infant weight	0.394	6.387	(0.001, 0.001)	0.000***	Yes
Breastfeeding duration → Infant weight	-0.158	-1.840	(-0.326, 0.010)	0.066†	Marginal
Any formula before complementary feeding → Infant weight	-0.055	-0.641	(-0.626, 0.318)	0.522	
Maternal pre-pregnancy BMI → Infant weight	-0.017	-0.241	(-0.066, 0.051)	0.810	
Maternal gestational weight gain → Infant weight	-0.113	-2.287	(-0.051, -0.004)	0.022*	Yes
Infant plasma IGF-1 → Infant weight	0.065	1.009	(-0.136, 0.425)	0.313	
Infant plasma insulin-to-glucose ratio → Infant weight	0.126	2.674	(0.073, 0.476)	0.007**	Yes
Cumulative protein intake → Infant plasma IGF-1	0.402	2.231	(0.031, 0.482)	0.026*	Yes
Infant age group → Infant plasma IGF-1	-0.127	-0.723	(-0.591, 0.273)	0.47	
Infant sex (male) → Infant plasma IGF-1	-0.238	-2.974	(-0.521, -0.107)	0.003**	Yes
Cumulative protein intake → Infant plasma insulin-to-glucose ratio	-0.021	-0.134	(-0.213, 0.186)	0.893	
Infant age group → Infant plasma insulin-to-glucose ratio	-0.070	-0.439	(-0.484, 0.306)	0.660	
Infant sex (male) → Infant plasma insulin-to-glucose ratio	-0.037	-0.464	(-0.258, 0.160)	0.643	
Plasma BCAA → Infant plasma insulin-to-glucose ratio	0.154	1.736	(0.047, 0.783)	0.083†	Marginal
Plasma BCAA → Infant plasma IGF-1	0.162	1.961	(0.000, 0.761)	0.050*	Yes
Model for Infant BMI outcome					
Cumulative protein intake → Infant BMI	0.243	1.206	(-0.189, 0.795)	0.228	
Infant age group → Infant BMI	-0.396	-2.226	(-1.828, -0.116)	0.026*	Yes
Infant sex (male) → Infant BMI	-0.106	-1.094	(-0.765, 0.217)	0.274	
Infant birth weight → Infant BMI	0.324	4.461	(1.671, 4.29)	< 0.001***	Yes
Breastfeeding duration → Infant BMI	-0.066	-0.637	(-0.238, 0.121)	0.524	
Any formula before complementary feeding → Infant BMI	-0.021	-0.187	(-0.606, 0.501)	0.852	
Maternal pre-pregnancy BMI → Infant BMI	0.036	0.364	(-0.057, 0.083)	0.716	
Maternal gestational weight gain → Infant BMI	-0.118	-1.485	(-0.059, 0.008)	0.137	
Infant plasma IGF-1 → Infant BMI	-0.055	-0.676	(-0.418, 0.204)	0.499	
Infant plasma insulin-to-glucose ratio → Infant BMI	0.138	1.965	(0.001, 0.531)	0.049*	Yes
Cumulative protein intake → Infant plasma IGF-1	0.434	2.415	(0.052, 0.502)	0.016*	Yes
Infant age group → Infant plasma IGF-1	-0.170	-0.975	(-0.642, 0.215)	0.329	
Infant sex (male) → Infant plasma IGF-1	-0.250	-3.113	(-0.541, -0.123)	0.002**	Yes
Cumulative protein intake → Infant plasma insulin-to-glucose ratio	-0.040	-0.254	(-0.227, 0.175)	0.799	
Infant age group → Infant plasma insulin-to-glucose ratio	-0.054	-0.337	(-0.472, 0.333)	0.736	
Infant sex (male) → Infant plasma insulin-to-glucose ratio	-0.040	-0.501	(-0.262, 0.155)	0.617	
Plasma BCAA → Infant plasma insulin-to-glucose ratio	0.156	1.754	(-0.044, 0.787)	0.079†	Marginal
Plasma BCAA → Infant plasma IGF-1	0.169	2.040	(0.015, 0.777)	0.041*	Yes

Supplementary Note. Example R workflow for the structural equation model (SEM)

Two SEMs were fitted in the study; here we show the full R workflow for the model in which infant body weight is the primary outcome. This example R script (i) loads required packages, (ii) screens distributional assumptions, (iii) specifies and fits the SEM, (iv) displays parameter estimates, and (v) checks key regression assumptions using a combination of linear models and linear mixed-effects models.

```
###########################################################################################
# 1.  LOAD PACKAGES
###########################################################################################
library(lavaan)      # SEM
library(MVN)         # normality tests
library(DHARMa)  # model assumption checks
library(lme4)        # model assumption checks
library(knitr)        # kable() for displaying formatted table


###########################################################################################
# 2.  DISTRIBUTIONAL ASSUMPTION SCREENING
###########################################################################################
# Robust ML handles non-normality, but we check normality assumptions and outliers up front.
mvn_result <- mvn(Data[ , c("IGF1", "Weight", "Age_group", "Male", "Birth_weight",
                              "Breastfeeding_duration", "Any_formula",
                             "Mother_prepregnancy_BMI", "Mother_gestational_weight_gain",
                             "Cumulative_protein_intake", "BCAA", "Insulin_to_glucose")],
                  mvn_test = "mardia")
# Inspect mvn_result for multivariate and univariate normality check results.


###########################################################################################
# 3.  SEM model specification and fitting
###########################################################################################
model <- '
 # Weight regressed on cumulative protein intake, adjusting for covariates
 Weight ~ Cumulative_protein_intake + Age_group + Male + Birth_weight + Breastfeeding_duration + Any_formula +
          Mother_prepregnancy_BMI + Mother_gestational_weight_gain

 # Weight regressed on IGF-1, adjusting for the same covariates
 Weight ~ IGF1 + Age_group + Male + Birth_weight + Breastfeeding_duration + Any_formula +
          Mother_prepregnancy_BMI + Mother_gestational_weight_gain

 # Weight regressed on insulin-to-glucose ratio, adjusting for the same covariates
 Weight ~ Insulin_to_glucose + Age_group + Male + Birth_weight + Breastfeeding_duration + Any_formula +
          Mother_prepregnancy_BMI + Mother_gestational_weight_gain

 # IGF-1 and insulin-to-glucose each regressed on cumulative protein intake, adjusting for covariates
 IGF1               ~ Cumulative_protein_intake + Age_group + Male
 Insulin_to_glucose ~ Cumulative_protein_intake + Age_group + Male

 # IGF-1 and insulin-to-glucose each regressed on BCAA, adjusting for covariates
 Insulin_to_glucose ~ BCAA + Age_group + Male
 IGF1               ~ BCAA + Age_group + Male
'
```

```r
fit <- sem(model, data = Data,
           estimator = "MLR",          # robust maximum likelihood estimation
           cluster   = "Subject_ID"))  # accounts for repeated measures per infant
# Clustering gives robust SEs, controlling for non-independence.

summary(fit,
        standardized = TRUE,      # prints Std.all coefficients
        fit.measures = TRUE)      # prints χ2, CFI, RMSEA, SRMR, etc.


##########################################################################################
# 4.  EXTRACT PARAMETER ESTIMATES
##########################################################################################
pe <- parameterEstimates(fit, standardized = TRUE, ci = TRUE, level = 0.95)
pe_subset <- pe[ , c("lhs", "op", "rhs", "std.all", "z", "ci.lower", "ci.upper", "pvalue")]

kable(pe_subset, digits = 3, caption = "Parameter Estimates from the SEM")


##########################################################################################
# 5.  ASSUMPTION CHECKS
##########################################################################################
# Goal:
#   • Re-fit each regression component with lmer()/lm()
#   • Use DHARMa to simulate residuals and visualize diagnostics

Model1 <- lmer(Weight ~ Cumulative_protein_intake + Age_group + Male + Birth_weight + Any_formula +
               Breastfeeding_duration + Mother_prepregnancy_BMI + Mother_gestational_weight_gain +
               (1 | Subject_ID), data = Data)

Model2 <- lmer(Weight ~ IGF1 + Age_group + Male + Birth_weight +  Any_formula + Breastfeeding_duration +
               Mother_prepregnancy_BMI + Mother_gestational_weight_gain +
               (1 | Subject_ID), data = Data)

Model3 <- lmer(Weight ~ Insulin_to_glucose + Age_group + Male + Birth_weight + Any_formula +
               Breastfeeding_duration +  Mother_prepregnancy_BMI + Mother_gestational_weight_gain +
               (1 | Subject_ID), data = Data)


Model4 <- lm(IGF1                ~ Cumulative_protein_intake + Age_group + Male, data = Data)

Model5 <- lm(Insulin_to_glucose ~ Cumulative_protein_intake + Age_group + Male, data = Data)


Model6 <- lmer(Insulin_to_glucose ~ BCAA + Age_group + Male + (1 | Subject_ID), data = Data)

Model7 <- lmer(IGF1                ~  BCAA + Age_group + Male + (1 | Subject_ID), data = Data)

# Simulate residuals and plot diagnostics

Assumption.check1 <- simulateResiduals(Model1);  plot(Assumption.check1)
Assumption.check2 <- simulateResiduals(Model2);  plot(Assumption.check2)
Assumption.check3 <- simulateResiduals(Model3);  plot(Assumption.check3)
Assumption.check4 <- simulateResiduals(Model4);  plot(Assumption.check4)
Assumption.check5 <- simulateResiduals(Model5);  plot(Assumption.check5)
Assumption.check6 <- simulateResiduals(Model6);  plot(Assumption.check6)
Assumption.check7 <- simulateResiduals(Model7);  plot(Assumption.check7)
```
